# Effect of Light-Emitting Diode Phototherapy on Serum Calcium Levels in Neonates With Jaundice

**DOI:** 10.7759/cureus.23938

**Published:** 2022-04-07

**Authors:** Karthikeyan Panneerselvam, Sathyamoorthy Mani, Nandini Vasudevan, Preethi S, Nedunchelian Krishnamoorthy, Pratibha RK, Subash Sundar

**Affiliations:** 1 Pediatrics, SRM Medical College Hospital and Research Centre/SRM Institute of Science and Technology, Kattankulathur, IND; 2 Preventive Medicine, SRM Medical College Hospital and Research Centre/SRM Institute of Science and Technology, Kattankulathur, IND; 3 Research, Mehta Multispeciality Hospital Pvt. Ltd., Chennai, IND

**Keywords:** hypocalcemia, phototherapy, light emitting diode, hyperbilirubinemia, term neonates

## Abstract

Background: To assess the change in serum total calcium levels during light-emitting diode phototherapy treatment for jaundice in term neonates.

Methods: A prospective observational study was done on 104 term neonates with hyperbilirubinemia in a tertiary care center to investigate the effects of phototherapy using a light-emitting diode device. The total serum bilirubin along with total calcium levels was measured at the start and at the end of phototherapy. Additionally, all the newborns enrolled in the study were evaluated for hypocalcaemia-related symptoms such as jitteriness, irritability/excitability, lethargy, and convulsions.

Results: A significant lowering of posttreatment total calcium level compared to that of pretreatment level (p<0.001) was found in our study. Hypocalcemia (serum calcium <8 mg/dL in term neonates) was found in 12.5% of the study subjects.

Conclusions: In the treatment of neonatal jaundice, similar to conventional blue and white light phototherapy, light-emitting diode phototherapy also has hypocalcemia as an adverse effect. We recommend monitoring these babies for hypocalcemia during light-emitting diode phototherapy.

## Introduction

Neonatal jaundice is a common issue during the newborn period. Preterm and term babies develop jaundice in their first week of life at a rate of approximately 80% and 60%, respectively [1]. Untreated unconjugated hyperbilirubinemia can cause neurotoxicity if not treated. Phototherapy is commonly used for managing neonatal hyperbilirubinemia. The duration of phototherapy is based on the gestational age of the newborn, hours of life after birth, and serum bilirubin level as per the American Academy of Pediatrics (AAP) guidelines chart [2]. Although phototherapy is a safer treatment modality, it does have some adverse effects, such as dehydration fluid loss, hyperthermia, rashes on exposed areas, loose stools, damage to the retina, bronze baby syndrome, hypocalcemia, and toxicity to genitalia [3].

Hypocalcemia is one of the common adverse effects of phototherapy. Hypocalcemia can be defined as either total serum calcium less than 8 mg/dL or ionized calcium less than 4.4 mg/dL in term or preterm neonates having a birth weight of more than 1500 g and as total serum calcium less than 7 mg/dL or ionized calcium less than 4 mg/dL in very low birth weight babies [4]. Some of the major symptoms of hypocalcemia are apnea, reduced feeding, cyanosis, vomiting, laryngospasm, cardiac failure, tachycardia, prolonged QT interval, tetany, irritability, jitteriness, and seizures [4].

Transcranial illumination during phototherapy probably blocks the cortisol effect on bone calcium by decreasing the secretion of melatonin from the pineal gland resulting in hypocalcemia. A direct effect of cortisol on calcium is that it increases calcium uptake and also causes hypocalcemia [5].

Light-emitting diode (LED) is a newer type of light source that is power efficient, has a long life, and is portable with minimal heat production [6]. Previous studies which have been done to determine the change in serum calcium levels during phototherapy in neonatal jaundice have used conventional blue and white phototherapy devices. Our study objective was to determine the change in serum total calcium levels in term neonates with neonatal hyperbilirubinemia between pre and post phototherapy using a light-emitting diode (LED) phototherapy device.

## Materials and methods

A hospital-based prospective observational study was done in a tertiary care teaching hospital in Tamil Nadu for six months from Feb 2021 to Aug 2021. Approval for this study was obtained from SRM Medical College Hospital and Research Centre Ethics Committee prior to its commencement (Ethics Clearance No: 2366/IEC/2021).

Term neonates (more than 37 completed weeks of gestation to less than 42 weeks of gestation) with neonatal jaundice who require phototherapy as per AAP. Phototherapy guideline charts were considered to be included in the study. A total of 104 term neonates were included in the study and their baseline variables like birth weight (kg), gestational age (weeks), and days of life (hrs) were recorded. Neonates looking icteric on clinical examination were subjected to baseline serum bilirubin and serum total calcium estimation after obtaining informed consent from the parents. 

All newborns with jaundice onset within 24 hours of birth, perinatal asphyxia (Apgar score < 4 within the first minute after birth), sepsis, with a history of exchange transfusion and jaundice beyond two weeks of life, hypocalcemia prior to the start of phototherapy, hypoalbuminemia, congenital anomalies, infant of a diabetic mother, and with a maternal history of anticonvulsant therapy were excluded from the study. Relevant history and complete physical examination were recorded for all the neonates.

A repeat serum bilirubin and calcium levels were recorded at the end of phototherapy. As part of our clinical assessment, we evaluated the neonates for clinical signs of hypocalcemia such as rashes, loose stool, fever, dehydration, irritability, jitteriness, and convulsions.

Data collection

Laboratory values of serum bilirubin and total calcium were obtained at the initiation and end of phototherapy for each subject.

Statistical analysis

The descriptive statistical data were represented as mean (Standard Deviation) for continuous variables and frequencies (percentage) for categorical variables. Kolmogorov-Smirnov test results showed a set of data appears to be normally distributed (P>0.05). Paired t-test was used to compare the serum calcium and serum bilirubin levels before and after phototherapy. Analysis of statistical data was evaluated using IBM SPSS software for Windows, Version 26.0 (IBM Corp., Armonk, NY). A p-value of ≤ 0.05, was taken as statistically significant.

## Results

A total of 104 term neonates admitted to neonatal care units for LED phototherapy were assessed for hypocalcemia in this study. Among these, 63 (60.6%) were males and 41 (39.4%) were females. Study participants had a mean birth weight of 2.99±0.32 kg, mean gestational age of 38.06±1.02 weeks, and 64.04±10.25 mean hours of life on admission for phototherapy. The total mean duration of phototherapy was 55.04±17.34 hours (Table 1).

**Table 1:** Distribution of descriptive statistics of the study participants (N=104)

Variable	Mean±SD	95% CI
Birth weight (kg)	2.99±0.32	2.92-3.05
Gestational age (weeks)	38.06±1.02	37.86-38.26
Days of life (hrs)	64.04±10.25	62.07-66.01
Serum bilirubin start of phototherapy	16.20±1.53	15.91-16.49
Serum bilirubin at 24 hrs of phototherapy	14.81±1.51	14.52-15.10
Serum bilirubin at the end of phototherapy	11.54±0.85	11.37-11.70
Total duration of phototherapy (hrs)	55.04±17.34	51.71-58.37
Serum calcium at the start of phototherapy	9.15±0.81	8.99-9.31
Serum calcium at the end of phototherapy	8.82±0.93	8.64-8.99

The serum bilirubin level among neonates at the start, at 24 hrs, and at the end of the phototherapy was 16.20±1.53 mg/dL, 14.81±1.51 mg/dL, and 11.54±0.85 mg/dL respectively. In comparison to serum bilirubin levels before phototherapy, serum bilirubin levels decreased statistically significantly after phototherapy (p<0.001) (Table 2).

**Table 2:** Distribution of variables before and after phototherapy (N=104)

S.No	Variable	Before starting phototherapy (mg/dL)	At the end of phototherapy (mg/dL)	p-Value
1	Serum calcium	9.15±0.82	8.82±0.93	<0.001
2	Serum bilirubin	16.20±1.53	11.54±0.85	<0.001

**Figure 1:**
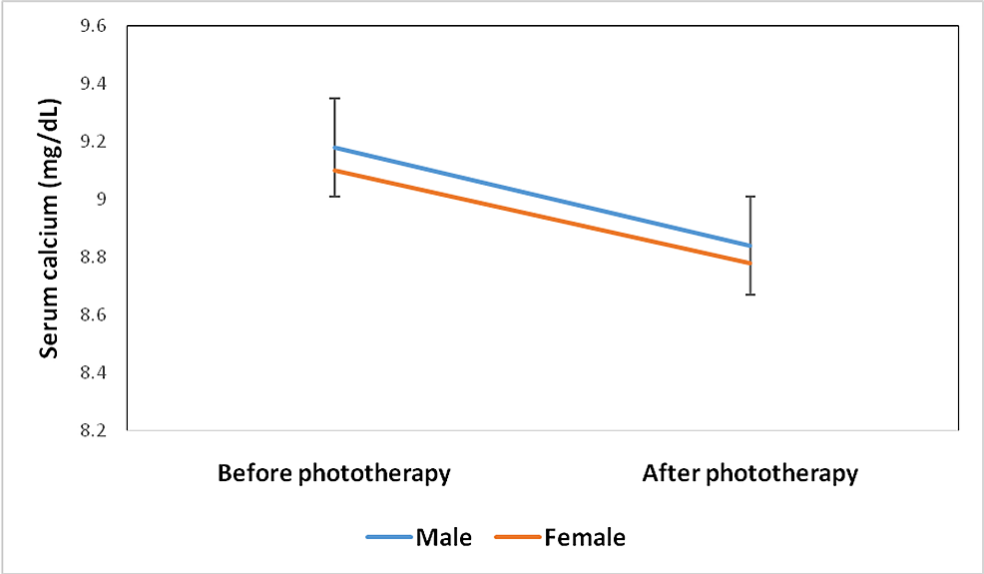
Distribution of serum calcium prior to and at the end of phototherapy with respect to gender (N=104)

The serum calcium levels for female babies were 9.10±0.74 mg/dL at the start of phototherapy and 8.78±0.96 mg/dL at the end of phototherapy. The serum calcium levels of male neonates were 9.18±0.86 mg/dL and 8.84±0.91 mg/dL at the start and the end of phototherapy respectively (Figure 1).

The serum calcium level among neonates at the start and end of the phototherapy was 9.15±0.81 mg/dL and 8.82±0.93 mg/dL respectively. The reduction in serum total calcium level after phototherapy was statistically significant (p<0.001) when compared to that before phototherapy (Table 2). Out of 104 study neonates, 13 (12.5%) developed hypocalcemia (total serum Ca < 8 mg%) at the end of LED phototherapy. But none of them showed clinical symptoms of hypocalcemia.

## Discussion

Phototherapy is a frequent and effective method for lowering indirect bilirubin levels in newborns. According to various literature reviews, hypocalcemia was found to be a common side effect of phototherapy when a standard blue and white phototherapy device is used. There are no previous studies that suggest a change in calcium levels following LED phototherapy.

The association of hypocalcemia with newborn phototherapy was first described by Romagnoli et al. [7]. Serum calcium level following phototherapy in our study was 8.82 + 0.93 mg/dL, which is closer to the reports published by Bahbah et al. (8.58 ± 0.76) [8] and similarly by Singh et al. (8.42 ±1.19) [3].

Alizadeh-Taheri et al. [9], Tehrani et al. [10], and Gheshmi et al. [11] reported phototherapy induced hypocalcemia in 7%, 7.5%, and 9% of neonates, respectively in their studies, which is lesser when compared to our study (12.5%). In a study by Karamifar et al., 14.4% of the newborns developed hypocalcemia, which is closer to our study (12.5%) [12].

Rajesh et al. [13] and Bahbah et al. [8] reported hypocalcemia in 26% of neonates whereas Shrivastav et al. [14] and Sethi et al. [15] showed hypocalcemia in 30% and 75%, respectively in their studies, which are higher when compared to our study. This higher incidence of hypocalcemia may be due to the inclusion of preterm babies in their study.

Among hypocalcemic neonates, 14% had jitteriness and 10% had convulsions in a study by Bahbah et al. [8], whereas 38% had jitteriness, and none of them developed convulsions in a study by Rajesh et al. [13]. None of the hypocalcemic newborns in our study showed clinical symptoms, which is consistent with the findings of Tehrani et al. [10] and Reddy et al. [16].

All of these studies have used blue and white phototherapy, whereas we used LED phototherapy. It should be mentioned that our study had several limitations, such as the exclusion of preterm infants, who are more likely to have hypocalcemia. During phototherapy, we did not measure ionized calcium levels. To estimate the proportion of hypocalcemia in jaundiced term infants undergoing LED phototherapy, we recommend larger multicentric studies to be carried out.

## Conclusions

Light-emitting diode phototherapy, like conventional blue and white light phototherapy, has a pontential to induce hypocalcemia as an adverse effect in the treatment of neonatal jaundice. During LED phototherapy, we urge that babies be monitored for hypocalcemia. Similar to various studies done using conventional blue and white light phototherapy, our study also does not support routine calcium supplementation for all babies undergoing LED phototherapy.
